# Correlations between preoperative statin treatment with short- and long-term survival following colorectal cancer surgery: a propensity score-matched national cohort study

**DOI:** 10.1007/s00384-024-04631-w

**Published:** 2024-04-27

**Authors:** Lea Löffler, Ismail Gögenur, Mikail Gögenur

**Affiliations:** 1 Center for Surgical Science, Zealand University Hospital, Lykkebækvej 1, 4600 Køge, Denmark; 2 Department of Clinical Medicine, University of Copenhagen, Blegdamsvej 3B, 2200 Copenhagen, Denmark; 3 Danish Colorectal Cancer Group, Copenhagen, Denmark

**Keywords:** Colorectal cancer, Statins, Surgery, Survival

## Abstract

**Introduction:**

The pleiotropic effects of statins have attracted considerable attention in oncological treatment. Several preclinical and epidemiological studies have highlighted their potential anti-tumor properties in patients with colorectal cancer, although results have been conflicting. This study aimed to examine the association between statin exposure before colorectal cancer surgery with long and short-term survival outcomes.

**Methods:**

This retrospective propensity score-adjusted study was conducted on a Danish cohort of patients who underwent elective curative-intended surgery for stage I–III colorectal cancer in 2008–2020, using four national patient databases. The primary and secondary outcomes were overall, 90-day, and disease-free survival. Propensity scores were calculated using all available data to match patients with and without statin exposure in a 1:1 ratio.

**Results:**

Following propensity score matching, 7120 patients were included in the primary analysis. The median follow-up time was 5 years. A Cox proportional hazards model showed no statistically significant difference in overall survival between patients with or without statin exposure 365 days before surgery (HR 0.93, 95% CI 0.85–1.02) and no association with 90-day survival (OR 0.91, 95% CI 0.76–1.10). However, a subgroup analysis examining a 90-day exposure before surgery found a statistically significant association with increased overall survival (HR 0.85, 95% CI 0.77–0.93).

**Conclusion:**

Although a subgroup of patients with a preoperative exposure time of 90 days showed statistically significant better overall survival, we found no statistically significant association between statin exposure 1 year before colorectal cancer surgery and overall survival.

**Supplementary Information:**

The online version contains supplementary material available at 10.1007/s00384-024-04631-w.

## Introduction

Colorectal cancer (CRC) is the third most prevalent cancer worldwide and the second leading cause of cancer-related deaths [1]. Furthermore, the incidence of early-onset CRC is increasing. Thus, the growing number of patients diagnosed with CRC presents a considerable global health challenge [2]. Surgical resection is the only curative treatment. However, this procedure is linked to a significant risk of postoperative complications [3], and despite surgery, many patients relapse, leading to cancer-related death [4].

The pleiotropic effects of 3-hydroxy-3-methylglutaryl-coenzyme A (HMG-CoA) reductase inhibitors, or statins, have gotten significant attention in oncological treatment in recent years. These drugs are widely used to treat dyslipidemia and to prevent cardiovascular disease [5]. Several preclinical and epidemiological studies have highlighted statins’ potential anti-tumor properties, including increased apoptosis [6, 7], reduced proliferation [8, 9], and reduced mortality [10, 11]. In addition, statins are believed to possess anti-inflammatory effects by inhibiting proinflammatory cytokines, such as IL-6, IL-8, and TNF-α [12]. These cytokines are typically released in response to surgical trauma. Thus, research has demonstrated an inverse correlation between the secretion of these cytokines and time to recovery [13, 14]. However, conflicting evidence about the protective effects of statins is evident in the literature, especially in cases of CRC [15, 16].

A Danish propensity score-matched cohort study that investigated statin exposure and short-term outcomes found no significant difference in 30-day survival between statin users and non-users in patients who underwent surgery for CRC [17]. Our hypothesis in this study was that statin exposure at any time 1 year before curative-intended surgery for CRC is associated with increased overall survival (OS) and 90-day survival. We aimed to investigate this in a nationwide propensity score-matched study.

## Methods

This study, employing retrospective propensity score adjustments, utilized a Danish patient cohort that underwent elective curative-intended surgery for Union of International Cancer Control (UICC) stage I–III CRC between 2008 and 2020. Patients who underwent emergency surgical procedures were excluded. The research adheres to the guidelines outlined in “Strengthening the Reporting of Observational Studies in Epidemiology” (STROBE) [18].

### Data sources

The data was sourced from the Danish Colorectal Cancer Group (DCCG), the Danish National Prescription Registry (NPR), the Danish Registry of Laboratory Results for Research (RLRR), and the Danish National Patient Registry (DNPR). The DCCG, established in 1994, constitutes a nationwide database encompassing patients aged 18 or above, possessing a Danish social security number, and diagnosed or surgically treated for primary CRC in a public Danish hospital from May 2001 onwards [19]. A recent validation of the database confirmed high completeness and quality [20]. Since 1994, comprehensive nationwide records of all prescribed medications have been registered in the NPR [21], while the RLRR has recorded individual biochemistry outcomes since 2008. These results originate from both private practitioners and public hospitals [22]. Lastly, established in 1977, the DNPR provides a comprehensive and longitudinal repository of administrative and clinical data at the national level. The database has recorded complete data encompassing treatments, diagnoses, and examinations across inpatient and outpatient clinics and emergency departments [23].

Data from the four databases were combined into a single Observational Medical Outcomes Partnership Common Data Model (OMOP CDM) through the utilization of open-source analytical tools created by the Observational Health Data Sciences and Informatics (OHDSI) community. The data extraction, transformation, and analysis process followed the principles of the OMOP [24].

### Study population

The study included all adults aged 18 or older who underwent curative-intent surgical procedures for UICC stage I–III CRC from 2008 to 2020. The target cohort consisted of patients who had been prescribed statins, and these prescriptions were active or in use during the period spanning up to 365 days before the surgical procedure took place, whereas patients in the comparator cohort were not exposed to statins. In case of subjects that are in both the target and comparator cohorts, the subjects were excluded. No sample size calculations were computed.

### Outcomes

The study’s primary outcome was to examine how preoperative statin exposure influences OS in patients having undergone curative intended surgery for UICC stage I–III CRC. OS was defined as the duration between the surgery to either death or the end of the 5-year follow-up period. The secondary outcomes were 90-day and disease-free survival. Disease-free survival analysis was performed in a cohort adhering to the criteria of the validated algorithm by Lash et al. [25]. In this analysis, time at risk is defined as 180 days following surgery until either recurrence, death, or end of follow-up. To assess the accuracy of the study design, five negative control outcomes were incorporated (Supplementary Table 1) [26].

### Subgroup analyses

The first subgroup analysis investigated the association between statin exposure of more or less than 30 mg/day 1 year before surgery and the primary and secondary outcomes.

The second and third subgroup analyses investigated the association between statin exposure between 30 and 90 days preoperatively and the primary and secondary outcomes.

Finally, two subgroup analyses were made for either rectal or colon cancer.

### Data security

This study obtained approval from the Danish Data Protection Agency under the registration number REG-102–2020. All data used in the research were anonymized and securely stored on the cloud-based platform Computerome 2.0, where all analyses were conducted.

### Statistical analysis

Using the Cyclops package for R [27], propensity score (PS) models were constructed for the primary and subgroup analyses. To create balanced cohorts, we employed a 1:1 matching ratio based on these propensity scores, which gauged the likelihood of a patient in the comparator group receiving statins compared to receiving no treatment. These scores considered a comprehensive dataset, including patient observations, demographic information, drug exposure, prior medical procedures, and medical history, spanning from any time before surgery as well as 2 years preceding surgery. Covariates linked to the treatment exposure were excluded (see Supplementary Table 2). Additionally, quantitative biochemistry data contributed to the propensity score calculation by considering the following criteria:

- Whether a specific analysis had been conducted for each patient
- The quantitative value of the biochemistry data
- Whether the biochemistry result fell within or outside the normal range

For missing records in the database, such as a missing diagnosis, we interpreted this as an absence of the diagnosis.

To match the patients 1:1, we employed a maximum caliper width of 0.2 standard deviation on the logit scale [28]. The evaluation of the PS model included a visual examination of Kernel density- and covariate balance scatter plots, along with the calculation of the standardized mean difference (SMD) for covariates. The predefined threshold for SMD difference was set at 0.1 in accordance with recommendations [29, 30]. Additionally, we conducted an unadjusted analysis in which the target and comparator cohorts were not matched.

To estimate the primary outcome, we utilized the Cox proportional hazards model, while a logistic regression model was employed for estimating the secondary outcomes. The results are presented as hazard ratios (HR) for the primary outcome analysis and odds ratios (OR) for the secondary outcome analyses, accompanied by 95% confidence intervals (CI). The proportional hazards assumption was evaluated to ascertain the constancy of hazard ratios over time with log–log survival plots and examination of Schoenfeld residuals. Cohorts were created using the open-source tool ATLAS, while analyses were carried through in R (version 4.2.0).

## Results

Six thousand four hundred fifty-one individuals who underwent elective surgery for CRC stage I–III between 2008 and 2020 with exposure to statins 365 days before the operation could be identified as the target group. The comparator cohort, consisting of patients not exposed to statins before surgery, comprised 20,477 individuals. After propensity score matching, both cohorts were reduced to 3560 patients each, resulting in a final analysis encompassing 7120 patients. The median follow-up time was 5.0 years (interquartile range [IQR] 2.6–5.0 years).

### Patient characteristics

On average and before propensity score matching, the patient group exposed to statins is older than the non-exposed group (SMD 0.12) and contains more males than females. Other notable differences in the exposed group are the higher percentage of patients in American Society of Anesthesiologists (ASA) group 3 (32.5% compared to 15.0%) as well as generally higher scores in the Charlson comorbidity index (see Table 1). These characteristics, as observed in our study, align with patterns identified in the general population of patients with colorectal cancer, as reported in the Danish Colorectal Cancer Group’s latest report from 2022 [31].

**Table 1:** Selected patient characteristics according to statin exposure before and after matching. Numbers in parentheses are percentages. The total for tumor localization differs from the cohort size due to synchronous cancer sites in the cohort

	Before propensity score matching			After propensity score matching		
Characteristics	Statin exposure	No statin exposure	SMD	Statin exposure	No statin exposure	SMD
*N*	6451	20,477		3560	3560	
Age, years						
≤ 49	44 (0.7)	967 (4.7)	− 0.25	32 (0.9)	32 (0.9)	0.00
50**–**74	3708 (57.5)	12,109 (59.1)	− 0.03	2038 (57.2)	1973 (55.4)	0.04
≥ 75	2699 (41.8)	7401 (36.2)	0.12	1490 (41.9)	1555 (43.7)	− 0.04
Sex						
Female	2542 (39.4)	9456 (46.2)	− 0.14	1595 (44.8)	1534 (43.1)	0.03
Male	3909 (60.6)	11,021 (53.8)	0.14	1965 (55.2)	2026 (56.9)	− 0.03
BMI, kg*m^**−**2^						
≤ 18.5	87 (1.3)	584 (2.9)	− 0.10	57 (1.6)	72 (2.0)	− 0.03
> 18.5 to ≤ 25.0	1927 (29.9)	7707 (37.7)	− 0.16	1146 (32.2)	1128 (31.7)	0.01
> 25.0 to ≤ 30.0	2264 (35.1)	5807 (28.4)	0.15	1194 (33.5)	1183 (33.2)	0.01
> 30.0 to ≤ 35.0	925 (14.3)	1765 (8.6)	0.18	438 (12.3)	447 (12.6)	− 0.01
> 35.0	342 (5.3)	595 (2.9)	0.12	152 (4.3)	171 (4.8)	− 0.03
Missing	906 (14.1)	4624 (22.6)	–	573 (16.1)	559 (15.7)	–
Charlson comorbidity index						
0	2745 (42.5)	15,576 (76.1)	− 0.73	1996 (56.1)	1899 (53.3)	0.05
1	1706 (26.4)	2560 (12.5)	0.36	803 (22.6)	847 (23.8)	− 0.03
2	997 (15.5)	1467 (7.2)	0.26	416 (11.7)	447 (12.6)	− 0.03
3	993 (15.4)	852 (4.2)	0.39	343 (9.6)	357 (10.0)	− 0.01
Missing	10 (0.2)	22 (0.1)	–	< 6 (0.0)	10 (0.3)	–
ECOG performance status						
0	1137 (17.6)	3177 (15.5)	0.06	588 (16.5)	603 (16.9)	− 0.01
1	706 (10.9)	813 (4.0)	0.27	295 (8.3)	283 (7.9)	0.01
2	208 (3.2)	220 (1.1)	0.15	81 (2.3)	84 (2.4)	− 0.01
3	45 (0.7)	54 (0.3)	0.06	15 (0.4)	18 (0.5)	− 0.01
4	8 (0.1)	8 (0.0)	0.03	< 6 (0.0)	< 6 (0.0)	0.03
Missing	4347 (67.5)	16,205 (79.1)	–	2581 (72.5)	2572 (72.2)	–
ASA						
1	426 (6.6)	5892 (28.8)	− 0.61	359 (10.1)	307 (8.6)	0.05
2	3654 (56.6)	10,699 (52.2)	0.09	2106 (59.2)	2135 (60.0)	− 0.02
3	2099 (32.5)	3073 (15.0)	0.42	949 (26.7)	983 (27.6)	− 0.02
4	139 (2.2)	196 (1.9)	0.10	65 (1.8)	53 (1.5)	0.03
5	< 6 (0.0)	< 6 (0.0)	0.01	< 6 (0.0)	< 6 (0.0)	0.01
Missing	133 (2.1)	617 (3.0)	–	81 (2.3)	82 (2.3)	–
Pathological UICC stage						
I	1518 (23.5)	4116 (20.1)	− 0.08	813 (22.8)	790 (22.2)	0.01
II	2662 (41.3)	8734 (42.7)	− 0.03	1473 (41.4)	1503 (42.2)	− 0.02
III	2147 (33.3)	7223 (35.3)	0.04	1201 (33.7)	1195 (33.6)	0.00
Missing	124 (1.9)	404 (1.9)	–	73 (2.1)	72 (2.0)	–
Tumor localization						
Rectum	2798 (43.3)	9537 (46.6)	− 0.06	1592 (44.7)	1523 (42.8)	0.04
Sigmoid colon	1395 (21.6)	4312 (21.0)	0.01	754 (21.2)	792 (22.2)	− 0.03
Descending colon and splenic flexure	350 (5.4)	941 (4.6)	0.04	190 (5.3)	204 (5.7)	− 0.02
Transverse colon	435 (6.6)	1154 (5.6)	0.05	220 (6.2)	214 (6.0)	0.01
Ascending colon and hepatic flexure	864 (13.3)	2467 (12.0)	0.04	460 (12.9)	483 (13.6)	− 0.02
Cecum	633 (9.8)	2115 (10.2)	− 0.02	357 (10.0)	361 (10.1)	− 0.00
Synchronous cancer	24	49	–	13	17	–

*ASA* American Society of Anesthesiologists, *BMI* body mass index, *ECOG* Eastern Cooperative Oncology Group, *SMD* standardized mean difference, *UICC* Union for International Cancer Control

### Propensity score matching

**Fig. 1:**
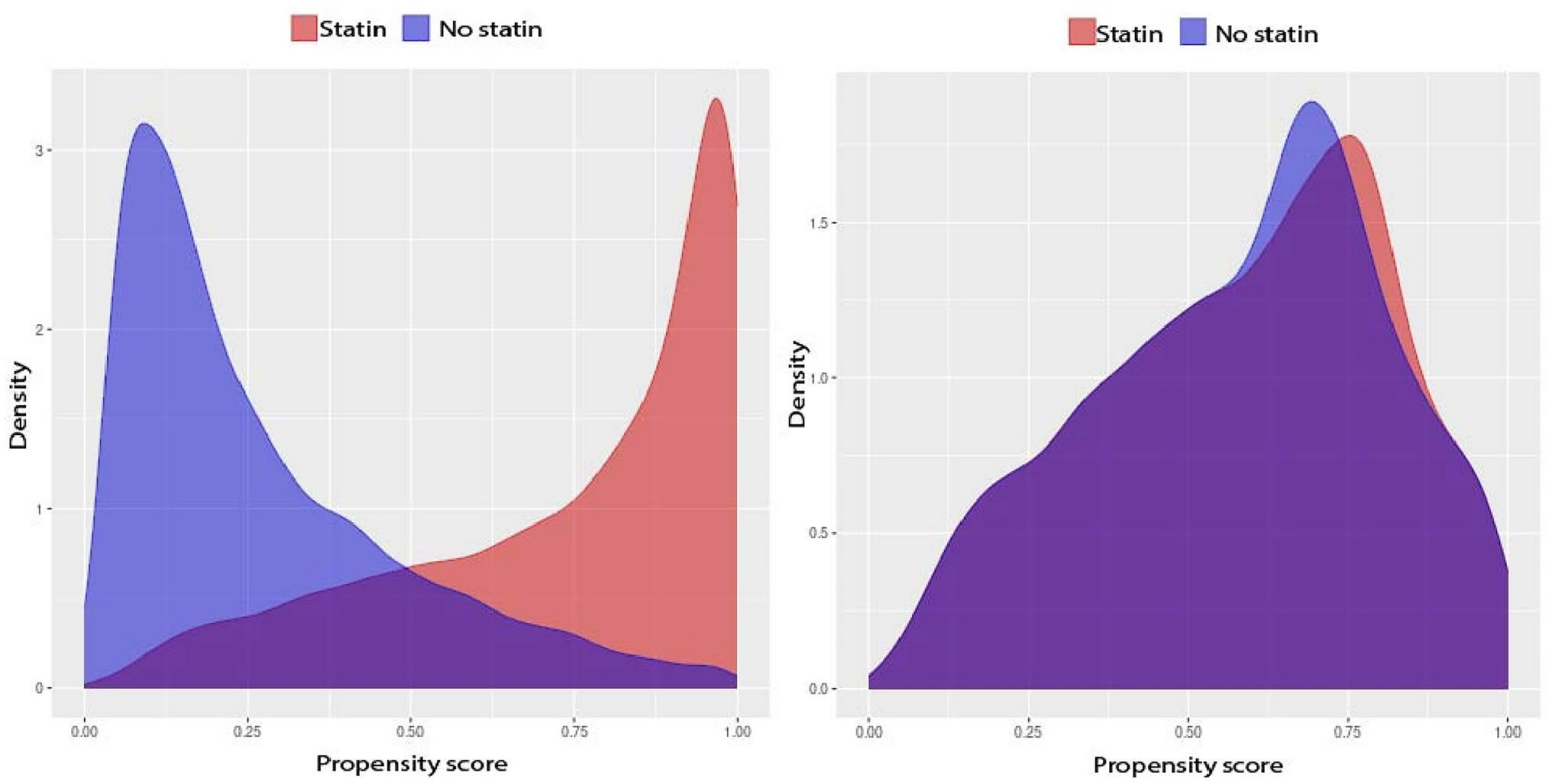
Distribution of patient propensity score in the main cohort before (left) and after propensity score matching (right)

Figure 1 shows the density plots of the propensity scores before and after matching.

The scatterplot illustrating the SMD before and after matching (Supplementary Fig. 1) indicates that 0.002% of the covariates (12 out of 61,231) exhibited SMD values below − 0.1 or above 0.1 following matching. Notably, the covariates showing the most substantial deviations from the threshold included the use of beta-blocking agents and thiazides within the 2 years preceding surgery, as well as at any time before the surgery.

### Overall survival

The unadjusted analysis that was performed of the groups before matching showed a statistically significant difference in OS between the patients exposed to statins 365 days before surgery versus the unexposed group (HR 1.06, 95% CI 1.01–1.12).

**Fig. 2:**
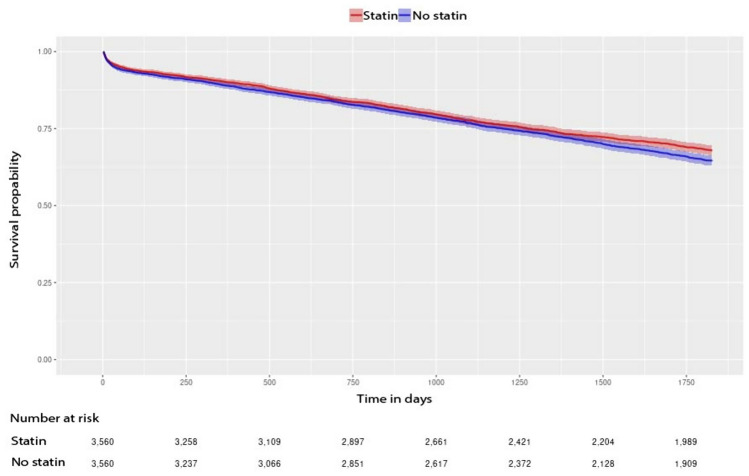
Overall survival in patients with colorectal cancer following surgery in the main adjusted analysis. Shown are Kaplan–Meier curves for patients with statin exposure 365 days preoperatively (red) versus no statin exposure (blue)

In the main analysis, the Cox PH model showed no statistically significant difference in OS between patients with statin exposure 365 days before surgery and those without statin exposure (HR 0.93, 95% CI 0.85–1.02) after PS matching (Fig. 2).

The log-log representation of survival trends and the evaluation of Schoenfeld residuals revealed no notable breaches of the assumption of proportional hazards.

**Fig. 3:**
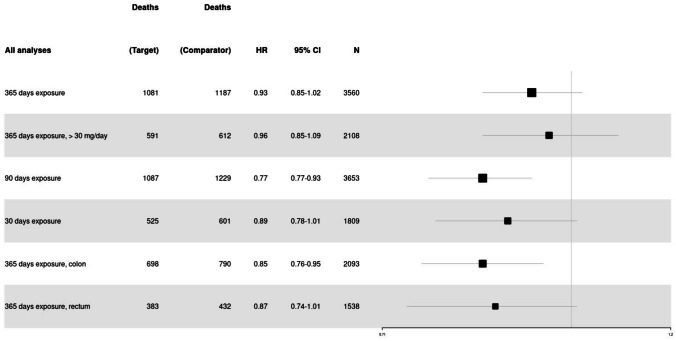
Forest plot of all analyses looking at overall survival. The number of patients (*N*) refers to the amount in one arm of the study. The results are only shown for the target group and not the comparator group that looked at no exposure to statins. The size of the black cube representing the HR varies proportionally based on the number of patients included in the specific analysis

The subgroup analysis examining a 90-day exposure to statins before surgery found the target group to have a significantly higher OS than the control group (HR 0.85, 95% CI 0.77–0.93). The subgroup analysis examining a 30-day exposure of statins before surgery (HR 0.89, 95% CI 0.78–1.01) as well as a statin dose of > 30 mg/day as opposed to ≤ 30 mg/day, 365 days preoperatively (HR 0.96, 95% CI 0.85–1.09), did not find a significant association with OS. The subanalysis investigating an association in the rectum cancer group found no significant association (HR 0.87, 95% CI 0.74–1.01). However, the colon cancer subgroup found the cohort exposed to statins to have a significantly higher OS (HR 0.85, 95% CI 0.76–0.95) (see Fig. 3).

### Ninety-day survival

**Fig. 4:**
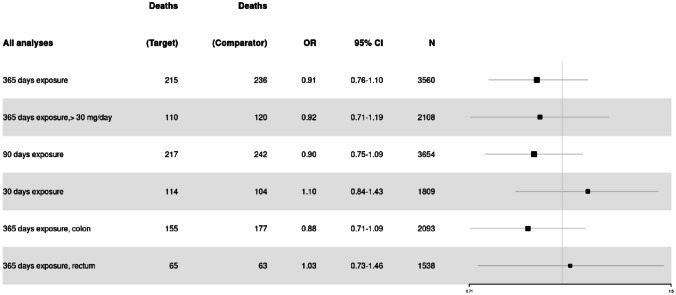
Forest plot of all analyses looking at 90-day survival. The number of patients (*N*) refers to the amount in one arm of the study. The results are only shown for the target group and not the comparator group that looked at no exposure to statins. The size of the black cube representing the OR varies proportionally based on the number of patients included in the specific analysis

The logistic regression model showed no statistically significant difference in 90-day survival between patients with statin exposure 365 days before surgery versus no statin exposure before surgery (OR 0.91, 95% CI 0.76–1.10). The subgroup analyses examining a 90- and 30-day exposure of statins before surgery did not find a significant association with 90-day survival (OR 0.90, 95% CI 0.75–1.08, and OR 1.10, 95% CI 0.84–1.43, respectively). Furthermore, there was no association in the subanalysis of > 30 mg statin/day 365 days preoperatively as opposed to ≤ 30 mg/day with 90-day survival (OR 0.92, 95% CI 0.71–1.19). The subanalyses looking into colon or rectal tumor only did not show any significant association (OR 0.88, 95% CI 0.71–1.09, and OR 1.03, 95% CI 0.73–1.46, respectively) (see Fig. 4).

### Disease-free survival

For the main analysis, the Cox PH model showed no statistically significant difference in DFS between patients with statin exposure 365 days before surgery and those without statin exposure (HR 0.93, 95% CI 0.84–1.03) after PS matching.

## Discussion

This study investigated the association between statin exposure and OS in patients undergoing elective colorectal cancer surgery in 2008–2020. The primary analysis of this study found no significant association between statin exposure at any time 365 days before surgery and improved OS. However, a subgroup analysis revealed a significant association when the exposure period of statins was 90 days before surgery. While the unadjusted analysis indicates a higher risk of death for patients exposed to statins, the propensity score-matched analysis shows a statistically significant improvement in OS for patients exposed to statins, accounting for potential confounding variables. There was no association with short-term survival.

The study’s findings add to the notion that statins may improve OS. While our study does not provide conclusive evidence to recommend the use of statins within 90 days prior to surgery, the observed positive association with overall survival suggests a potential avenue for further exploration, for example, through translational research. Investigating specific signaling pathways, immune modulation, and relevant biological processes could help uncover the mechanisms through which statins influence the disease. Additionally, studying changes in immune cell populations, cytokine profiles, and response dynamics within the tumor could provide insights into how statins contribute to a more favorable environment for overall survival. This could pave the way for a more comprehensive understanding and potentially enhance the clinical relevance of statins in the context of colorectal cancer management. However, the protective effects of statins in patients with CRC are still debated, and results are conflicting. While Poynter et al. found a 47% decrease in the relative risk (RR) of the development of CRC when exposed to statins for a period of 5 years [11], a meta-analysis that included 18 studies reviewed the preventive effects of statins in patients with CRC and suggested that there is no substantial evidence to support this claim. Nevertheless, a slight reduction of risk cannot be ruled out [15]. A different meta-study reviewed both the preventive role of statins in CRC patients and its role in adjuvant therapy, concluding that statins may decrease the invasiveness or metastatic properties of CRC as well as sensitizing the tumor to chemotherapeutic agents. However, further research is needed to assess this hypothesis [16]. A Swedish retrospective study [32] reported a statistically significant lower 90-day survival in patients with colon cancer, whereas our study found no significant association between statin exposure and 90-day survival in this same subgroup. Our study used the propensity score matching method, while the Swedish study chose covariates for matching, including sex, age, BMI, cancer stage, and surgical details. Both studies observed that statin users tend to be older and have more comorbidities. Notably, the Swedish study had a larger sample size (6494 patients) than ours (3560 patients). These differences in study design and sample size could contribute to the divergent results and highlight the need for further investigation in the context of colon cancer treatment and statin therapy. In general, study designs and patient characteristics in all studies above were heterogeneous, thus increasing variability in study outcomes. Another aspect that could explain the conflicting evidence regarding the protective effect of statins in patients with CRC is patient compliance as well as the COVID-19 pandemic.

Studies suggest that statins may have protective effects against severe COVID-19 outcomes by mitigating inflammation, highlighting their multifaceted potential in the context of both colorectal cancer and infectious diseases like COVID-19 [33, 34].

This study’s strengths lie in its use of nationwide population-based databases within the OMOP-CDM framework, enabling detailed propensity score calculation. Matching based on these scores minimizes confounding, approximating the randomized controlled trial (RCT) gold standard [29]. National Danish registry data collection is uniform and comprehensive, reducing selection and attrition bias.

Adopting OHDSI’s open-source tools establishes a standardized framework for observational health research, enhancing reproducibility, transparency, and data consistency.

However, unaccounted covariates in the CDM, such as over-the-counter medications, behavior, and lifestyle, can introduce residual confounding. Further potential issues include missing data, misregistration, and the inability to assess statin compliance in this model. While our study did not explicitly account for socioeconomic factors, it is essential to contextualize our findings within the broader landscape highlighted by previous research. Notably, low socioeconomic position (SEP), especially low income, consistently associates with statin non-adherence, as evidenced by Danish and Finnish studies [35, 36]—both countries with universal healthcare—conducted on data from 1995 to 2004. These investigations explored the correlation between SEP and statin utilization, revealing a socioeconomic gradient in statin use among men. Intriguingly, no such clear association was observed among women, suggesting potential gender-specific variations in the influence of socioeconomic factors on medication use. Although these studies utilize older data, their findings emphasize the enduring relevance of socioeconomic status as a potential confounder in studies evaluating statin effectiveness. The identified socioeconomic gradient among men underscores the necessity of considering SEP when interpreting and generalizing results from studies assessing the impact of statins on health outcomes.

In conclusion, this study found no significant association between statin exposure within 365 days before surgery and improved OS in patients with CRC. A subgroup analysis investigating a 90-day exposure before surgery found a significant association, suggesting that statins may have a potential role in the survival of patients with CRC. Further research is needed to understand statins’ effects on CRC patients better— conducting translational analyses to delve into molecular mechanisms, immune modulation, and patient-specific factors could thus hold the potential to offer valuable insights, laying the groundwork for future clinical applicability.

## Supplementary Information

Below is the link to the electronic supplementary material.

Supplementary file1 (DOCX 55 KB)

## Data Availability

The sharing of source data is prohibited under Danish legislation, as it includes sensitive personal information.
