# Supplementary material for: Correlations between preoperative statin treatment with short- and long-term survival following colorectal cancer surgery: a propensity score-matched national cohort study
Source: Int J Colorectal Dis. 2024 Apr 27;39(1):60. doi: 10.1007/s00384-024-04631-w (PMC11055774; doi:10.1007/s00384-024-04631-w)

**Appendices**

*Supplementary Table 1 – Negative controls, all analyses*

| **ID** | **Description** | **Domain** |
| --- | --- | --- |
| 194133 | Low back pain | Condition |
| 201620 | Kidney stone | Condition |
| 4344500 | Impingement syndrome of shoulder region | Condition |
| 4308093 | Dupuytren’s disease of palm | Condition |
| 380094 | Carpal tunnel syndrome | Condition |

*Supplementary Table 2 – Variables that were excluded in the propensity score matching analysis*

| **ID** | **Description** | **Domain** |
| --- | --- | --- |
| 1539403 | Simvastatin | Drug |
| 1510813 | Rosuvastatin | Drug |
| 1551860 | Pravastatin | Drug |
| 922570 | Nystatin | Drug |
| 1592085 | Iovastatin | Drug |
| 1549686 | Fluvastatin | Drug |
| 1592180 | Cerivastatin | Drug |
| 1545958 | Atorvastatin | Drug |

*Supplementary Figure 1 – Standard Mean Difference after propensity score matching, overall survival main analysis. The SMD value of each covariate before and after matching is represented by a blue dot, main analysis*


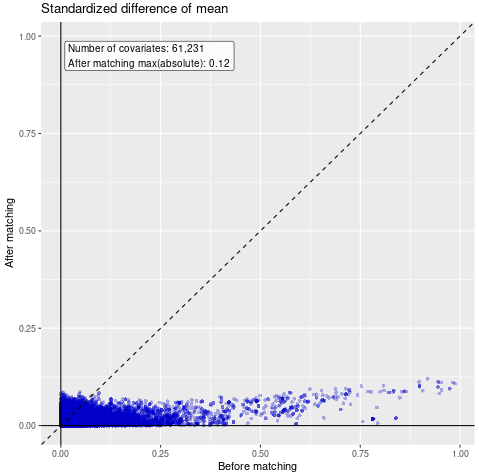

Supplement: Supplementary file 1 — Supplementary file1 (DOCX 55 KB) [file 384_2024_4631_MOESM1_ESM.docx]
